# New trajectories or accelerating change? Zooarchaeological evidence for Roman transformation of animal husbandry in Northern Italy

**DOI:** 10.1007/s12520-020-01251-7

**Published:** 2021-01-15

**Authors:** Angela Trentacoste, Ariadna Nieto-Espinet, Silvia Guimarães, Barbara Wilkens, Gabriella Petrucci, Silvia Valenzuela-Lamas

**Affiliations:** 1 Institute of Archaeology, University of Oxford, Oxford, UK; 2 Consejo Superior de Investigaciones Científicas (CSIC), Institució Milà i Fontanals, Archaeology of Social Dynamics, Barcelona, Spain; 3 CIBIO-InBIO, Centro de Investigação em Biodiversidade e Recursos Genéticos, Universidade do Porto, Vairão, Portugal; 4 Alghero, Italy; 5 Trieste, Italy

**Keywords:** Agriculture, Improvement, Economy, Biometry, Iron Age, Roman Empire, Late Antiquity

## Abstract

Throughout the Western provinces of the Roman Empire, greater economic and political connectivity had a major impact on agricultural production, which grew in scale and specialisation after integration with the Roman state. However, uniquely in Western Europe, farming strategies in Italy began to evolve centuries before the Roman conquest, and many ‘Roman’ patterns associated with livestock size and the relative proportions of different taxa first emerged during the early and middle centuries of the first millennium BC. These changes imply a significant reorganisation of production strategies well before Roman hegemony, even in relatively marginal areas of Italy. Zooarchaeological studies have documented further significant changes to livestock production in Roman times, but the relationship between these developments and earlier trends remains unclear. Through analysis of zooarchaeological data for species representation and livestock biometry from lowland northern Italy (Po–Friulian Plain), this study investigates animal exploitation between the Bronze Age and Late Antiquity in order to characterise the influence of Roman political and economic organisation on animal husbandry. Results demonstrated subregional variation in species representation, and different trajectories in the biometric evolution of cattle, sheep and goats, compared to pigs. Initial steps established in the Iron Age towards a more complex and dynamic livestock economy were accelerated and further reconfigured in Roman times, facilitated by Roman economic organisation and the specialised and large-scale production systems within it. Zooarchaeological trends continued to progress over the Roman period, until further changes at the very end of the chronology considered here—around the sixth century AD—suggest another wave of change.

**Supplementary Information:**

The online version contains supplementary material available at 10.1007/s12520-020-01251-7.

## Introduction

Political unification under the Roman Empire had a profound impact on the social and economic organisation of Western Europe, and, consequently, the agricultural strategies employed throughout this territory. Greater connectivity brought new tastes (Rowan 2019), and wider exchange networks transformed food production, allowing greater specialisation and the long-distance transport of an agricultural surplus (Mattingly and Aldrete 2000; Ward-Perkins 2006). New plants and animals were introduced (Witcher 2013; Bosi et al. 2020), and both rare species and common foods circulated with greater intensity (Orengo and Livarda 2016). Although different regions followed distinct trajectories, zooarchaeological studies document a suite of changes in livestock production across the Western Empire. Firstly, changes in the relative proportions of livestock illustrate the evolution of animal husbandry regimes and suggest change in meat preferences (e.g. King 1999; Valenzuela-Lamas and Albarella 2017). Secondly, distinct modes of carcass processing associated with Roman towns and military establishments became recognisable, and large structured animal bone deposits point to specialised processing in these contexts (e.g. Groot 2016; Rizzetto et al. 2017; Seetah 2018). Thirdly, after millennia of size diminution, livestock—especially cattle—increased significantly in size. In Western Europe, this increase in body size has been documented in Britain (Johnstone 2004; Albarella et al. 2008), France (Méniel 1984; Lepetz 1996; Forest and Rodet-Belarbi 2002; Frémondeau et al. 2017; Duval and Clavel 2018), Belgium (Pigière 2017), the Netherlands (Lauwerier 1988), Germany (Teichert 1984; Groot 2017), Switzerland (Breuer et al. 1999; Groot and Deschler-Erb 2015, 2017), the Balearic Islands (Valenzuela et al. 2017) and the Iberian peninsula (Altuna 1980; Colominas and Saña 2009; Colominas 2013; Colominas et al. 2017). However, within continental-scale trends, evidence of diversions and different regional rhythms are also found: Portugal (Valenzuela-Lamas and Detry 2017; Nieto-Espinet et al. 2021) and Rhaetia (Trixl et al. 2017) show little change in livestock size after the Roman conquest. Zooarchaeological evidence also demonstrates that this reorganisation of animal production, although profound, was not permanent: in many areas changes to species representation, carcass processing and animal size relaxed or reversed over Late Antiquity and the early Middle Ages (e.g. Rizzetto et al. 2017; Duval and Clavel 2018; Salvadori 2019).

Livestock improvement in Western Europe has traditionally been viewed as the product of ‘Roman’ husbandry strategies related to improved feeding practices (e.g. Kron 2002), changes in sex ratios with greater preference for male animals (Forest and Rodet-Belarbi 2002), and the importation of new phenotypically larger stock (e.g. Méniel and Brunaux 1983; Méniel 1996; Minniti et al. 2014; Colominas and Edwards 2017). Recent research, however, has revealed an increase in livestock size prior to Roman influence in some areas of Western Europe. In northern Gaul, increases are visible from as early as the third century BC (Duval et al. 2012; Frémondeau et al. 2017), during a period in which an expansion in trade networks and changes in territorial and administrative structure are attested (Buchsenschutz 2015; Duval et al. 2018). These changes in animal size then accelerated during Roman times. Furthermore, uniquely in Western Europe, livestock in Italy increased significantly in size even earlier, during the early and middle centuries of the first millennium BC (De Grossi and Minniti 2017; Valenzuela-Lamas and Albarella 2017; Trentacoste et al. 2018; De Grossi and Minniti 2019). Over a similar period, a pig-focused subsistence strategy emerged in parts of central and northern Italy (De Grossi and Minniti 2009; Minniti 2012; Trentacoste 2016). As in the Gallic examples, these changes to Italian animal management were probably catalysed by the development of new economic strategies and social relationships, evidenced archaeologically by a reorganisation of settlement networks and new forms of connectivity and human mobility (see Fulminante 2014; Antonio et al. 2019; Cavazzuti et al. 2019a; Cavazzuti et al. 2019b). These zooarchaeological trends then intensified in Roman times: further increases in livestock size and an even greater reliance on pork consumption are recorded in Imperial Italy (MacKinnon 2004a, 2010; Love 2008; De Grossi and Minniti 2017). Are these Roman trends an acceleration of pre-existing tendencies, or do they represent a new direction in animal management?

**Fig. 1:**
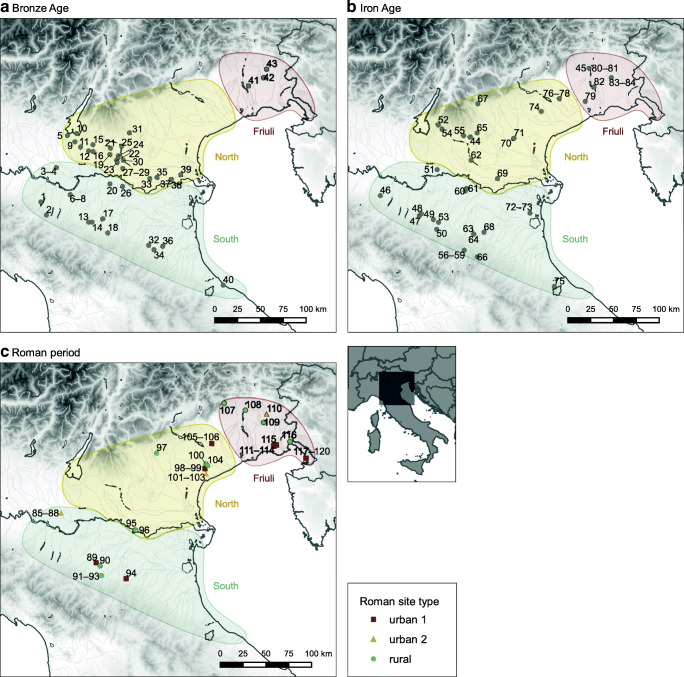
Map of sites showing location of the three study regions. See Online Supplement 1 for site details. Terrain data from the U.S. Geological Survey

An appreciation of the pace and character of Roman developments, and to what extent these differed from earlier protohistoric strategies, is essential to understanding the nature of agriculture in Roman Italy, and to what extent Roman developments represented technical innovations versus a reorganisation of existing strategies in a new socioeconomic context. These conclusions have implications for how we understand the impact of Roman political annexation on production, and how we conceptualise what defines ‘Roman’ food and farming. This study analyses zooarchaeological data for species representation and livestock biometry in order to investigate the influence of Roman sociopolitical and economic organisation on animal husbandry in lowland northern Italy (Fig. 1). The study area covers the eastern Po Valley and adjacent Venetian–Friulian Plain, encompassing an area of comparable climate and topography (see Rubel et al. 2017) within Cisalpine Gaul: an area later incorporated into Roman Italy as the Augustan regions of Venetia and Histria (regio X) and Aemilia (regio VIII). We expand previous studies of Roman animal biometry in northern Italy (e.g. Riedel 1994b; MacKinnon 2001, 2004a, 2010) with data published during the past decade to analyse a significant body of zooarchaeological information from an underrepresented region of Roman Italy. The primary focus is on the late Iron Age, Roman, and Late Antique periods, but results are placed in context over the *longue durée*, from the Middle Bronze Age through Late Antiquity (c. 1700 BC–AD 700), to understand how Roman developments relate to long-term trends. This analysis builds on a similar study dedicated to the Bronze and Iron Ages in the Po–Friulian Plain (Trentacoste et al. 2018); consequently, prehistory is not discussed in detail in this paper, although the comparative data provide an important point of comparison for the degree and pace of change. This investigation allows us to address to what extent Roman transformations to animal management represents the acceleration of established trends versus new directions in livestock husbandry, and it lays a foundation for future zooarchaeological, isotopic and genetic analyses in the region.

## Archaeological and historical context

Over the early part of the Iron Age (c. 950–early second century BC), material culture across the study area developed more distinct regional identities, leading to the emergence of proto- and then fully Etruscan, Venetian and Golaseccan traditions (Bonetto 2009; Bietti Sestieri 2010). Zooarchaeological evidence for increases in the size of sheep and cattle suggests a significant reorganisation of animal production from as early as the Bronze–Iron Age transition, well before Roman influence in the region (Trentacoste et al. 2018). Settlements and trade networks in the southern Po Plain and Veneto expanded over the early part of the first millennium BC, and by the fifth century BC cities with urban characteristics and site hierarchies had developed (Balista et al. 2002; Govi 2014). In the southern Po Plain, a new pig-focused subsistence strategy developed around the flourishing network of Etruscan cities (Trentacoste 2016, 2020). Livestock representation in other parts of lowland northern Italy demonstrates greater continuity or variability, and higher percentages of cattle remains (Trentacoste et al. 2018).

The fourth and third centuries BC saw a cultural and economic reorganisation of the territory, as the presence of La Tène material culture increased (Frey 1995; Williams 2001). Much of the central and western Po Plain became dominated by peoples of ‘Celtic’ origin (Curina et al. 2015). These migrations caused major disruption to the Etruscan urban network that occupied this area: settlement organisation became more fragmented and dispersed, and cities declined (Malnati 1988; Sassatelli and Govi 2013). Archaeobotanical evidence broadly suggests continuity in arable crop choice over the Iron Age (Trentacoste and Lodwick in prep.), although the greater diffusion of imported millstones from the fourth century BC (e.g. Renzulli et al. 2002) illustrates developments in the technology—and possibly also scale—of crop processing. Animal production also demonstrates a degree of continuity through the final centuries before Roman control of the region, although there are few assemblages from this period to offer a robust assessment. The Etruscan cities of Spina and Marzabotto continued to produce high frequencies of pig bones (47–59%) during the fourth–third centuries BC (Curci 2010; Briccola et al. 2013), although at lower percentages and possibly in different management systems than at their Archaic peak. Major transitions in animal management are not visible north of the River Po (Trentacoste et al. 2018).

The Roman Republic gained control of Cisalpine Gaul through campaigns over the third and early second centuries BC. The adjacent territories of Rhaetia and Noricum (eastern Switzerland, Tyrol, Austria) were later incorporated by Augustus during the first century BC (see Smith 2017; Roncaglia 2018 for recent summaries). Roman political control had a profound impact on the territory, initially through the foundation of colonies and construction of roads (Laurence 1999; Matteazzi 2017). Life in the region was also reshaped by immigration and integration with Roman economic and social networks (Curina et al. 2015)—developments supported by the extension of citizenship to communities south of the River Po (89 BC), and later to *Transpadana* north of the river (49 BC). With the creation of *regiones* by Augustus, the area was incorporated into Roman *Italia*. Archaeological evidence demonstrates limited rural occupation before the second century BC, with greater in-filling of the landscape during the first centuries BC and AD (Calzolari et al. 2003; De Franceschini 2003; Matteazzi 2014, 2017). This was also a period of significant territorial organisation via centuriation, land reclamation, and development of a new water management network (Calzolari et al. 2003; Bruno et al. 2013; Matteazzi 2016, 2017; Cremaschi et al. 2018). A broad survey of zooarchaeological evidence from across Italy has suggested that the first century BC was also an important point of change in animal management (Trentacoste and Lodwick in prep.), which became more organised and integrated into Roman trade networks at moment of significant economic expansion (Wilson 2014). The evolution of animal production strategies during the early Roman transitional period is difficult to assess due to the paucity of assemblages dated exclusive between the late third to early first century BC.

During Imperial times (first–third centuries AD), intensive land exploitation in northern Italy led to significant deforestation (Marchetti 2002; Bosi et al. 2011). Human activity shaped a landscape in which cereal fields and pasture were punctuated by gardens, orchards, and thin woodland, alongside freshwater marshy areas (Bosi et al. 2011; Bosi et al. 2015; Bosi et al. 2019). Botanical remains demonstrate arable cultivation focused on wheats and barley alongside millets, and the consumption of various legumes and new types of fruits, raised both in fields and garden plots (Bosi et al. 2020). Agricultural strategies also impacted livestock, and biometric studies have demonstrated that cattle in northern Italy became even larger during the Roman period (Riedel 1994b; MacKinnon 2004a, 2010). However, in Alpine areas of northern Italy, cattle demonstrate greater continuity in size (MacKinnon 2010; Trixl et al. 2017). Together with the rural landscape, the built environment was also reshaped, as urban centres constructed the hallmark buildings of Roman life (theatres, bathes, temples, basilicas) and villas were built in their rural territories (De Franceschini 2003).

Beginning in the third century AD and escalating in the fourth–fifth centuries AD, communities in northern Italy were profoundly impacted by invasions, plague and political power struggles surrounding imperial succession (Christie 2006; Roncaglia 2018). A reorganisation of the countryside, during the late third and especially fourth century AD, is evidenced by a reduction in rural sites, and the degradation of the roads and land preservation systems (De Franceschini 2003; Fontana et al. 2008; Cremonini et al. 2013; Matteazzi 2017). However, while overall numbers of rural sites declined in the fourth century, many surviving villas were rebuilt, some at larger sizes and elaborately refurbished: changes that point to the growth of large estates and consolidation of land holding in the hands of elites (Sfameni 2004). Urban areas reduced in size, and forests expanded (Bosi et al. 2015; Bosi et al. 2019). Political instability led to greater militarisation of towns and surrounding landscape, reflected both in the construction of walls and other fortifications, as well as a reconfiguration of the regional economy, as state-owned supplying the army operated from northern Italian towns (James 1988; Christie 2006).

Following the disposition of the last Western emperor in AD 476, Gothic rulers of Late Antique Italy largely continued the established economic system (Castagnetti 1982; Ward-Perkins 2006), until the Gothic Wars and subsequent political fragmentation of the peninsula brought about an end to *romanitas* in northern Italy (Marazzi 1997). Alongside this social and political division, the Justinianic Plague and climatic cooling during the Late Antique Little Ice Age (Whittow 2019) contributed to a collapse of the agrarian system (Banaji 2012; Castrorao Barba 2014; Forin 2017). Without maintenance of land reclamation systems, climatic deterioration during the sixth century AD (McCormick et al. 2012; Bruno et al. 2013) caused large areas of the Po Plain to become marshland, at least periodically (Marchetti 2002; Brandolini and Cremaschi 2018; Bosi et al. 2019). Nonetheless, many ‘Roman’ towns continued to be inhabited into medieval times, even if in a form structurally, politically, and economically very different than during earlier periods (La Rocca 1992; Ward-Perkins 1997; Christie 2006).

This reconfiguration of the urban and rural landscape over Late Antiquity had an impact on agricultural production, although dramatic changes do not appear to have been immediate. Regional zooarchaeological studies found further increases in cattle and pig biometry during Late Antiquity (MacKinnon 2001, 2004a, 2010), suggesting that any changes to production impacted animal size after this period. Archaeobotanical evidence from northern Italy suggests continuity in crop choice, with a focus on barley and naked wheats, but with greater diversification of local resources from the fifth–sixth centuries AD (Squatriti 2013; Rottoli 2014). When Italy is considered as a whole, zooarchaeological studies suggest general continuity until around the sixth century AD, when a decrease in the proportion of cattle remains, body size reductions, and changes in mortality patterns indicate a break from Roman and Late Antique production systems (Salvadori 2011, 2015, 2019). In cattle, this reduction in relative abundance and gradual decrease in height reached a minimum in the tenth–eleventh centuries AD (Riedel 1994b; Salvadori 2019). In contrast, sheep and pigs followed appear to have remained more stable in height between the Roman period and Early Middle Ages (Salvadori 2015).

## Materials and methods

### Sites and assemblages

Sites considered in this metadata analysis were selected from the eastern Po Valley and the Venetian–Friulian Plain (Online Supplement 1). Materials associated with religious or cultic activity were excluded from NISP analyses. Ritual animal bone assemblages from Italy often have features distinct from those of habitation sites, with notable biases toward a particular species or body part (see Barker 1989; De Grossi and Minniti 2012; Trentacoste 2016). This pattern of selective killing and deposit curation continues from prehistory into the Roman period (e.g. De Grossi and Minniti 2002; De Grossi 2004; Corbino and Fonzo 2017), thus warranting separate consideration of ritual and habitation assemblages when species frequencies are compared. Where possible, contextual details were considered, and surface finds and plow zone materials excluded. Assemblages were assigned to a chronological period: Bronze Age (c. 1650–950 BC), Iron Age (including the Etruscan period, c. 950–180 BC), and Roman period/Late Antiquity (c. 180 BC to 7th century AD) (see Online Supplement 1). The Iron Age, Roman, and Late Antique periods were further subdivided into subphases, which were considered in greater detail: IA1 (8th to 6th century BC), IA2 (6th to 2nd century BC), Early Roman (Republican and early Imperial: 2nd century BC to 1st century AD, including assemblages dated within the 1st century AD), Mid Roman (Imperial: 1st to 3rd century AD), and Late Roman (Late Antiquity: 4th to 7th century AD). The Bronze Age and earliest centuries of the Iron Age (tenth to eighth centuries BC) were investigated in detail in a previous paper (Trentacoste et al. 2018). Assemblages with long chronologies between the 1st and 5th centuries AD were designated to an intermediate Mid–Late phase. Roman/Late Antique materials that could not be confidently assigned to one of the above phases were classified ‘General’. Roman sites were classified following MacKinnon (2004a:32–33) as rural (villas, rural settlements), urban 2 (sites with urban characteristics not confirmed as *municipia*), and urban 1 (urban sites known to be *municipia)* (see Fig. 1c) to compare differences in livestock abundance between different site types.

### Analysis of livestock abundance and biometry

The number of identified specimens (NISP) was used to quantify the relative abundance of cattle (*Bos taurus*), sheep/goat (*Ovis aries/Capra hircus*), and pigs (*Sus domesticus*). Although a widely employed method for investigating taxon representation, NISP is subject to systematic biases related to specimen interdependence, fragmentation and inter-observer variation in identification skill and recording practices (Grayson 1979; Lyman 1994; Morin et al. 2016). To better control for these concerns, skeletons and articulating bone groups were excluded from NISP counts. Rib and vertebrae fragments, which were identified to species only in some reports, were also excluded. Only assemblages with more than 100 remains were considered in quantification of taxon abundance. Differences in species abundance were tested with a chi square test.

Animal size and morphology were investigated using log standard index (LSI) values (Simpson et al. 1960; Meadow 1999) and direct comparison of measurements (in millimetres) from mandibular third molars (M3s) and metacarpals. LSI values from width and length measurements were calculated following the method presented in Trentacoste et al. (2018). This method employs one measurement per specimen and one bone from any group of articulating remains in order to avoid over representation of particular individuals. Published standards for cattle (Nieto-Espinet 2018), sheep/goat (Davis 1996) and pigs (Albarella and Payne 2005) were used to calculate LSI values of post-cranial bones. Measurements from the mandibular third molar (M3) were used to investigate tooth biometry. Teeth, and especially tooth widths, are thought to be less susceptible to environmental changes than post-cranial bones (Payne and Bull 1988). Changes in sex ratios were investigated using measurements from metacarpals. The lower front limb is sexually dimorphic in domestic bovids (Higham 1969; Thomas 1988; Davis 2000), and is also thought to be so in suids (Payne and Bull 1988). Consequently, these elements can be used to identify male and female animals from archaeological remains (Davis et al. 2012). To facilitate interpretation, metacarpal biometry of cattle was compared to archaeological specimens of known sex from Eketorp (Telldahl et al. 2012), a ringfort in Sweden dated c. 300–1250 AD, and Beja, a 15th-century AD site in Portugal (Davis et al. 2012); metacarpal measurements from sheep were compared to those of a modern Shetland flock (Davis 1996, 2000).

Statistical differences in LSI values between major periods and phases with the Iron Age and Roman period were analysed using Mann–Whitney *U* tests. Confidence intervals (95%) for M3 measurements were calculated based on a multivariate *t* distribution using the stat_ellipse function in the R package ggplot2.

## Results

### Taxon abundance

**Fig. 2:**
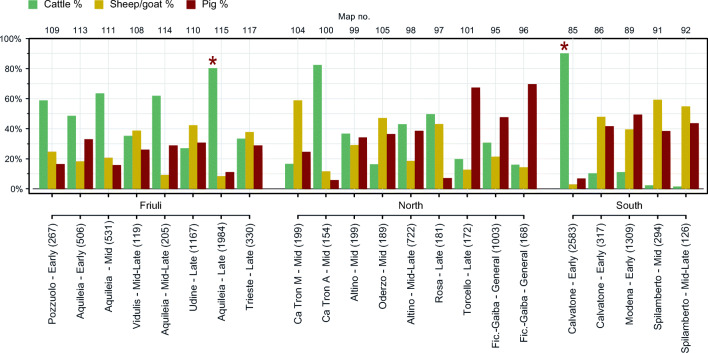
Relative proportions of livestock on Roman sites. NISP in parenthesis. Stars indicate NISP > 1500. See Online Supplement 1 for details

Livestock representation varied across Roman and Late Antique sites (Fig. 2; Online Supplement 1). Cattle tended to predominate in Friuli, a pattern driven by their abundance in assemblages from Aquileia. Assemblages from the southern Po Plain generally contained higher proportions of sheep/goats and pigs and low (< 12%) percentages of cattle, with the exception of one assemblage from Calvatone. Livestock ratios from the North study area were more variable, with some regional trends. The two assemblages from Ficarolo-Gaiba, on the southern border of the region, had high percentages of pigs, followed by cattle. Torcello—an island the Venetian lagoon—had a similar distribution of taxa. Taxon frequencies in other assemblages in the North region were very diverse.

**Fig. 3:**
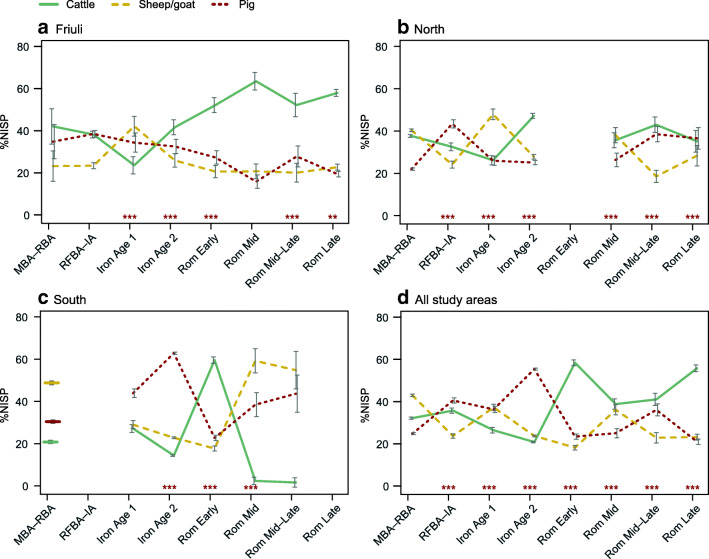
Relative proportions of livestock by region and period. MBA–RBA: Middle–Recent Bronze Age. RFBA–IA: Recent/Final Bronze Age–Early Iron Age. Iron Age 1: 8th–6th century BC. Iron Age 2: 6th–2nd century BC. Asterisks indicate significant differences between phases (*p* < 0.01**, *p* < 0.001***). See Table 1 for details on NISP and number of assemblages. See Online Supplement 3 for results of chi square tests

Assemblages differed significantly in their nature and size, which influenced regional trends: the two largest assemblages, from Aquileia and Calvatone (indicated in Fig. 2), each had a livestock NISP over 1500, which included a very high proportion of cattle remains, as well as distinct patterns in skeletal element distribution and evidence of relatively standardised butchery. At a regional level, these large assemblages had a clear impact on broader patterns in NISP trends (Table 1, Fig. 3). This was especially evident in the South study area, where the exceptional cattle-dominated assemblage from Calvatone rendered the Early Roman phase markedly different from adjacent phases (Fig. 3c). NISP percentages for the large deposit from Aquileia were comparable to other late Republican and Imperial assemblages from the same city. These two large assemblages also influenced species representation on different site types (Online Supplement 2): when all rural and urban assemblages were compared, urban assemblages contained significantly higher proportions of cattle remains. However, when the two largest assemblages from Aquileia and Calvatone were excluded, livestock representation was relatively balanced between the three taxa across different site types, although with a lower proportion of cattle and greater representation of sheep/goat at urban 2 sites.

**Table 1:** NISP and relative percentages of livestock by region and period. See Online Supplement 1 for details

	Bronze Age–Iron Age				Roman and Late Antique phases				
	Middle–Recent BA	Recent/Final BA–Early IA	Iron Age 1: 8th–6th c. BC	Iron Age 2:6th–2nd c. BC	Early	Mid	Mid–Late	Late	General
Friuli									
NISP	138	3536	437	769	773	531	324	3481	
Assemblages	1	4	1	2	2	1	2	3	
Cattle	42%	38%	24%	42%	52%	63%	52%	58%	
Sheep/goat	23%	23%	42%	26%	21%	21%	20%	23%	
Pig	35%	38%	34%	33%	27%	16%	28%	19%	
South									
NISP	10883		2322	32701	4209	294	126		
Assemblages	14		7	11	3	1	1		
Cattle	21%		27%	14%	60%	2%	2%		
Sheep/goat	49%		29%	23%	18%	59%	55%		
Pig	30%		44%	63%	23%	38%	44%		
North									
NISP	22394	2607	1606	7435		741	722	353	1171
Assemblages	17	7	1	8		4	1	2	2
Cattle	38%	32%	26%	47%		36%	43%	35%	29%
Sheep/goat	40%	24%	48%	28%		38%	19%	28%	20%
Pig	22%	43%	26%	25%		26%	39%	37%	51%
All									
NISP	33415	6143	4365	40905	4982	1566	1172	3834	1171
Assemblages	32	11	9	21	5	6	4	5	2
Cattle	32%	36%	26%	21%	58%	39%	41%	56%	29%
Sheep/goat	43%	24%	37%	24%	18%	36%	23%	23%	20%
Pig	25%	41%	36%	55%	23%	25%	36%	21%	51%

Sheep were more abundant than goats on all Roman/Late Antique sites where these taxa were identified to species level. The ratio of sheep to goats was comparable across the study areas, although the dataset was small: South (2.8, *n* = 19), North (3, *n* = 32) and Friuli (2.03, *n* = 97). These ratios varied across Roman/Late Antique phases: Early (2.8, *n* = 19), Mid (1.7, *n* = 70) and Late (2.8, *n* = 53).

Diachronic changes in species representation differed across the study areas. When Northern Italy is considered as a whole (Fig. 3d), the relative abundance of cattle decreases over prehistory, before increasing significantly in the Early Roman phase. Cattle remained the most abundant taxon for the remainder of the Roman/Late Antique period, although at lower percentages than documented at the Early Roman peak. Trends in sheep/goats and pigs were more variable. All differences in NISP at the macro-regional level were significant according to chi square tests (*p* < 0.001) (Online Supplement 3).

Within these macro-regional trends, the three study areas followed distinct trajectories (Fig. 3a–c). In both Friuli and the northern Po Plain, the relative abundance of cattle decreased over the Bronze Age and early part of the Iron Age, before increasing in the late Iron Age. In Friuli, proportions of cattle continued to increase in Roman phases, and they remained the most abundant taxon across the Roman and Late Antique periods. Livestock representation in the North study area in Roman times was more balanced, if variable, with a modest increase in the relative proportion of pigs and decrease in the abundance of sheep/goats in later Roman phases. The South study area registered very high frequencies of pigs in the Iron Age (i.e. Etruscan period), while cattle generally decreased in abundance over the time period considered, with a notable exception in the Early Roman period due to the large cattle dominated assemblage from Calvatone. Diachronic changes in NISP relevant to Iron Age and Roman phases were statistically significant in all instances, except the transition from the Early to Mid Roman period in Friuli, and from the Mid to Mid–Late periods in the southern Po Plain (see Online Supplement 3).

### Biometry

Summary statistics for LSI values and measurements from M3s from major periods are presented in Online Supplement 4 and 5. Summary statistics for LSI values from Roman phases are shown in Online Supplement 6. The results of Mann–Whitney *U* tests comparing LSI values and tooth measurements from different periods are found in Online Supplement 7; results for phases within the Iron Age and Roman periods are presented in Online Supplement 8.

#### Cattle

Analysis of LSI values from cattle post-cranial bones demonstrated a progressive increase in both width and length LSI values across major chronological periods (Fig. 4.1). These changes were highly significant in all comparisons (*p* < 0.001). Measurements from cattle teeth also registered an increase in size between major periods (Fig. 4.2); again, all differences were highly significant (*p* < 0.001). Comparison of length and width measurements from individual teeth (in mm) showed that the size increase in teeth occurred along a similar regression line (Fig 4.2c). Comparison of LSI values from phases within the Iron Age and Roman periods (Fig. 5) revealed significant changes in length values between IA2 and Early Roman phases (*p* > 0.001). Significant differences in width values were also found between IA2 and Early Roman phases (*p* > 0.001), as well as between Mid and Late (p = 0.020) phases, and Mid–Late and Late (*p* < 0.001) phases.

**Fig. 4:**
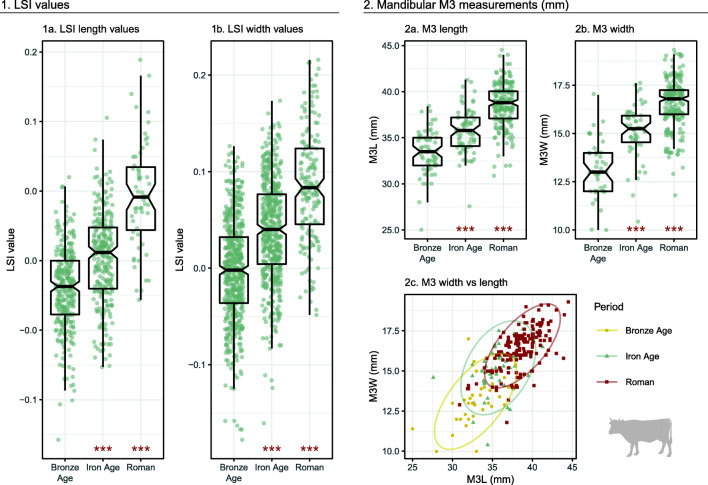
Cattle LSI values and mandibular third molar (M3) measurements by period. Asterisks indicate significant diachronic changes according to Mann–Whitney *U* tests: *p* < 0.001***, *p* < 0.01**, *p* < 0.05* (see Online Supplement 7). Ellipses in Fig. 4.2c represent 95% confidence intervals

**Fig. 5:**
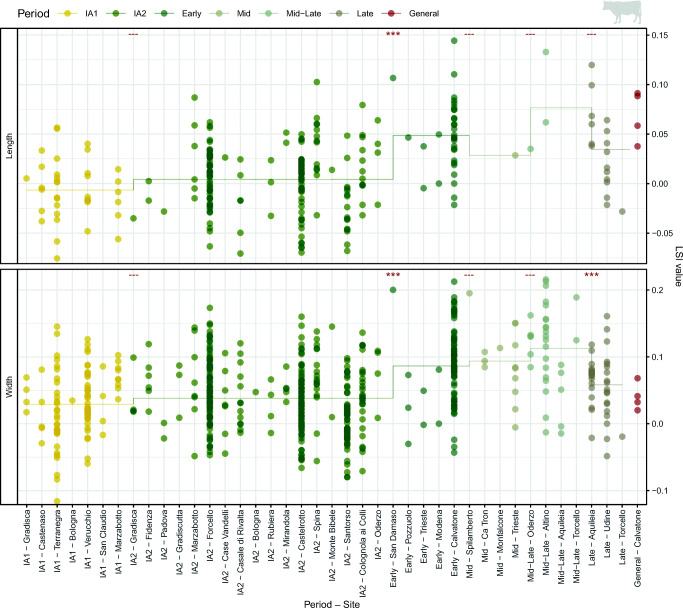
Cattle LSI values from Iron Age, Roman and Late Antique sites. Asterisks indicate significant diachronic changes according to Mann–Whitney *U* tests: *p* < 0.001***, *p* < 0.01**, *p* < 0.05* (see Online Supplement 8). Horizontal line marks the phase mean

When compared with later prehistory, cattle metacarpal biometry (Online Supplement 9) illustrated the presence of large cattle during Roman times, similar in size and robustness to animals (especially males) from fifteenth-century Beja. Compared to later prehistory, metacarpals from small cattle (Bd <58 mm) were rare in Roman/Late Antique times. The distribution of distal metacarpal breadth measurements from the Roman/Late Antique did not produce a clear bimodal distribution, complicating assessment of sex ratios; however, the limited data occupy a similar range to the mixed male–female population at Beja.

#### Sheep/goat

**Fig. 6:**
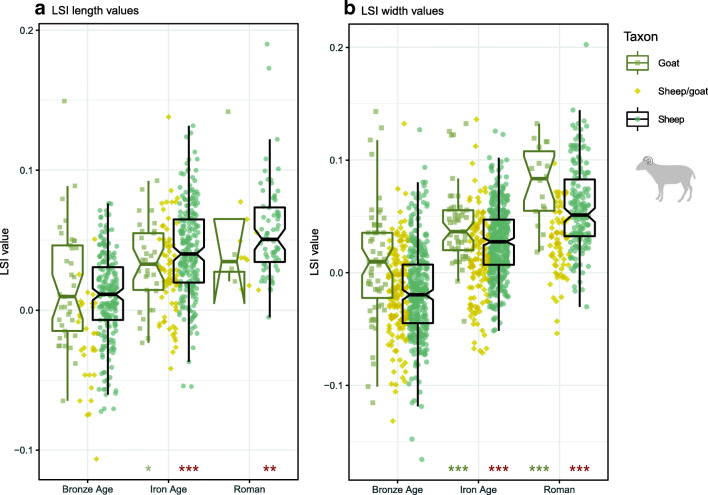
Sheep, sheep/goat and goat LSI values by period. Box plots only displayed for sheep and goat. Asterisks indicate significant diachronic changes according to Mann–Whitney *U* tests for sheep (red) and goats (grey): *p* < 0.001***, *p* < 0.01**, *p* < 0.05* (see Online Supplement 7)

Sheep, goat and sheep/goat LSI values demonstrated an increase across major periods (Fig. 6). In sheep, this size increase was statistically significant for both length and width values. Most inter-period comparisons had highly significant results (*p* < 0.001), while the significance of change between the Iron Age and Roman/Late Antique period was less strong in length values (*p* = 0.005). Although mean values increased, in LSI lengths this was primarily influenced by the lack of small values in the Roman period: the upper range of LSI length values was similar between the Iron Age and Roman times.

**Fig. 7:**
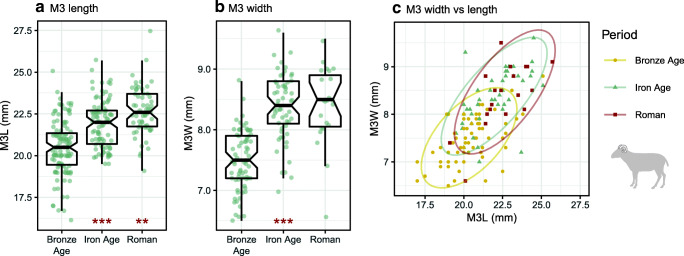
Sheep/goat mandibular third molar (M3) measurements by period. Asterisks indicate significant diachronic changes according to Mann–Whitney *U* tests for sheep (red) and goats (grey): *p* < 0.001***, *p* < 0.01**, *p* < 0.05 (see Online Supplement 7). Ellipses in Fig. 7c represent 95% confidence intervals

In goats, LSI width values followed a similar trend, with significant interperiod increases (*p* < 0.001). LSI length values from goats also registered an increase in later prehistory (*p* = 0.044), but changes in length values between the Iron Age and Roman period, for which there were few data (Roman *n* = 4), were not significant. Measurements from sheep/goat M3s corroborated trends visible in post-cranial LSI values (Fig. 7). M3 length increases were statistically significant between both the Bronze Age and Iron Age (*p* < 0.001) and Iron Age to Roman/Late Antique times (*p* = 0.001). Increases in M3 widths were significant over prehistory (*p* < 0.000), although no significant change was observed between the Iron Age and Roman period. The scatter plot of sheep/goat M3 measurements indicates that the size change in teeth occurred along similar regression lines, with similarity in tooth size between the Iron Age and Roman/Late Antique periods.

**Fig. 8:**
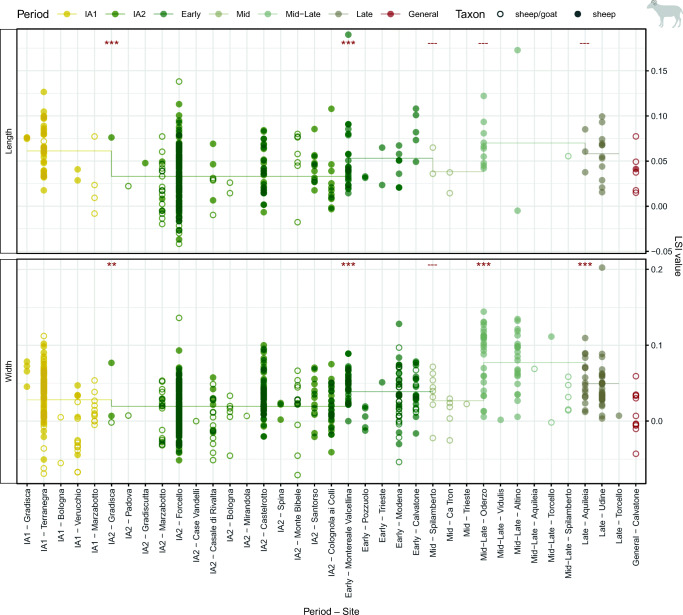
Sheep and sheep/goat LSI values from Iron Age, Roman and Late Antique sites. Asterisks indicate significant diachronic changes according to Mann–Whitney *U* tests: *p* < 0.001***, *p* < 0.01**, *p* < 0.05* (see Online Supplement 8). Horizontal line marks the phase mean

Within Iron Age and Roman/Late Antique phases (Fig. 8), significant diachronic differences in combined sheep and sheep/goat LSI length values were noted between the IA1 and IA2 phases (*p* < 0.001), as well as IA2 and Early Roman phases (*p* < 0.001). Analyses of width values produced significant differences between several phases: IA1 and IA2 (*p* = 0.002), IA2 and Early Roman (*p* < 0.001), Roman Mid and Mid–Late (*p* > 0.001), and Roman Mid–Late and Late (*p* > 0.001). When values from sheep were considered independently, similar results were produced (see Online Supplement 8)

Measurements from sheep metacarpals (Online Supplement 10) demonstrate diachronic changes in size and shape. Iron Age metacarpals were longer and more slender than those of the Roman/Late Antique period, which are more robust and comparable with Shetland rams. Within the Roman period, there appear to be two metacarpal groups: one very robust group, and a second more slender group similar to Iron Age individuals. This division is even more apparent in metacarpal distal breath (Bd) measurements: compared to the Bronze and Iron Ages, Roman/Late Antique Bd values produced a broad and clearly separated bimodal distribution.

#### Pigs

LSI results for pigs include large values from specimens identified as wild boar alongside a broad range of values from bones identified as domestic pigs or generally as ‘*Sus*’ (Fig. 9.1). Wild boar generally plotted as outliers, separated from the smaller domestic population by a gap around LSI value 0.05. Outliers in width values were more clearly separated from the main pig population than length outliers. When a relatively conservative value of 0.05 is used to separate large (potentially wild) from small (potentially domestic) pigs, LSI values record a decrease in the percentage of ‘large’ individuals between the Bronze Age and Iron Age, with relative stability in percentages between the Iron Age and Roman/Late Antique times (Table 2).

**Fig. 9:**
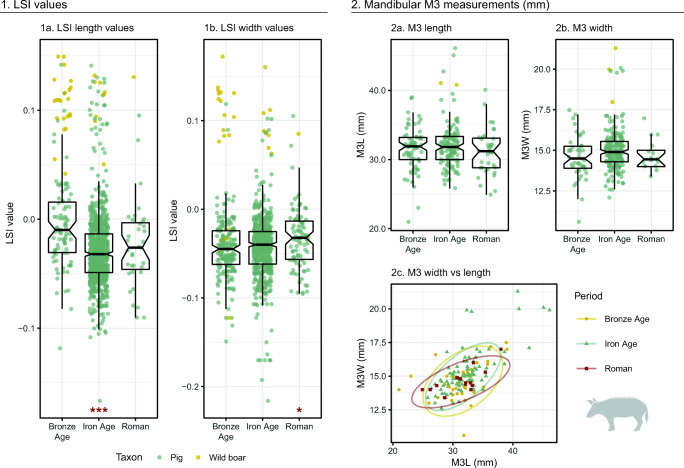
Pig and wild boar LSI values and mandibular third molar measurements by period. Box plots exclude specimens identified as wild boar. Asterisks in boxplots indicate significant diachronic changes according to Mann–Whitney *U* tests: *p* < 0.001***, *p* < 0.01**, *p* < 0.05* (see Online Supplement 7). Ellipses in Fig. 9.2c represent 95% confidence intervals

**Table 2:** Relative abundance of LSI values from pigs (all *Sus* sp.) in different size classes

	Bronze Age		Iron Age		Roman	
	Lengths	Widths	Lengths	Widths	Lengths	Widths
LSI value > 0.05 (wild?)	24%	8%	5%	3%	8%	3%
LSI value < 0.05 (domestic?)	76%	92%	95%	97%	92%	97%
*n*	115	212	777	571	36	91

Unlike domestic bovids, LSI values from pig post-cranial bones did not demonstrate a clear diachronic increase over later prehistory. In contrast, there was a significant decrease in length LSI values between the Bronze Age and Iron Age (*p* < 0.001), and no significant difference in late prehistoric width values. Between the Iron Age and Roman period, width values registered a weakly significant increase (*p* = 0.012), while length values did not produce a statistically significant change. Statistical tests produced similar results when large values comparable in size to wild boar (LSI value greater than 0.05) were excluded (lengths: Bronze Age–Iron Age *p* < 0.001, Iron Age–Roman *p* = 0.492; widths: Bronze Age–Iron Age *p* = 0.440, Iron Age–Roman *p* = 0.016). M3 widths and lengths registered a small increase in mean molar width between the Bronze and Iron Ages, followed by a decrease in Roman/Late Antique times (Fig. 9.2); however, these changes were small and not statistically significant. The scatterplot of pig M3 measurements also demonstrates relative continuity in tooth size between periods (Fig. 9.2c).

**Fig. 10:**
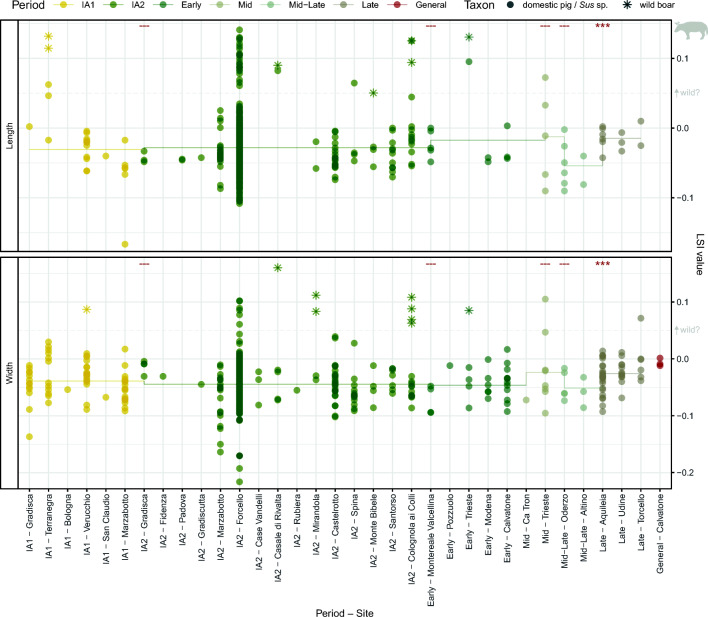
Pig and wild boar LSI values from Iron Age, Roman and Late Antique sites. Asterisks indicate significant diachronic changes in domestic pig/*Sus* sp. values according to Mann–Whitney *U* tests: *p* < 0.001***, *p* < 0.01**, *p* < 0.05* (see Online Supplement 8). Horizontal line marks the phase mean for values identified as domestic pig/*Sus* sp.

Within the Iron Age, Roman, and Late Antique phases, both LSI length and width values (Fig. 10) registered a weakly significant change between the Roman Mid–Late and Late phases (lengths: *p* = 0.005; widths: *p* = 0.031). When outliers greater than 0.05 were excluded, Mann–Whitney *U* tests produced similar results, and the only comparison found to be significant was between the Mid–Late and Late phases (lengths: *p* = 0.005; widths: *p* = 0.035). Measurements from pig metacarpals (Online Supplement 11), especially fourth metacarpals, suggest a notable diachronic change in *Sus* shape. Iron Age and especially Roman metacarpals tend to be markedly more robust than Bronze Age specimens, excluding bones larger than c. 85 mm in length (and thus probably from wild boar).

## Discussion

### Regional diversity in taxon abundance and new forms of animal processing

When northern Italy is considered as a single unit, it demonstrates a rise in cattle percentages during Roman times—an increase that is also documented in the neighbouring provinces of Germania, Rhaetia, and Noricum (King 2001; Deschler-Erb 2017; Trixl et al. 2017). Compared to central and southern Italy, our results confirm the pronounced inter-regional differences in livestock representation documented in previous studies of the Roman period (MacKinnon 2004a; Ikeguchi 2017). Cattle remains are more abundant in northern Italy than other parts of the peninsula, especially when compared to the pork-focused consumption strategies that define Rome and its hinterland (De Grossi and Minniti 2017). However, our results suggest that this cattle-dominant pattern was predominantly a feature of Friuli (particularly Aquileia) and some special assemblages, rather than a representation of northern Italy as a whole.

Analysis of taxonomic abundance demonstrated different subregional trajectories in livestock representation. In Friuli, the proportion of cattle rose across the Iron Age and into the Roman Imperial period, from IA1 to the Roman Mid phase. In the South study area, the preference for small livestock, i.e. sheep/goats and/or pigs, that predominated in Bronze Age and Etruscan assemblages continued into the Roman period, with the exception of a particular deposit from Calvatone. If this assemblage is excluded, the relative abundance of cattle in the region continued to fall from IA1, through IA2, and into Roman phases, with lower proportions of cattle in each of these periods compared to other contemporary study areas. The North region, however, demonstrated significant diachronic and intraperiod variability in taxon representation. This diversity appeared partly impacted by site locations and the different environmental situations that they represented. For instance, Torcello—the only island and one of the latest sites included in this study—contained a high proportion of pig remains. Assemblages from Ficarolo-Gaiba, a minor rural settlement, also had high pig percentages, which—given its location near the border with the South study region—may reflect the trends towards small livestock seen in assemblages south of the Po River.

While these trends are based on a small number of assemblages, sometimes only one or two for a region and time period, they nevertheless evidence complex subregional dynamics in NISP representation. When amalgamated at a regional level, these dynamics are obscured by a few large assemblages that reflect particular forms of cattle butchery. The two largest Roman assemblages also attest to specialised cattle processing: Calvatone (the vicus of *Bedriacum*) (Wilkens 1997) in the South study area, as well as the city of Aquileia in Friuli. At the latter site, assemblages associated with markets in the town’s centre produced characteristic ‘hook-marked’ scapulae associated with meat smoking and standardised butchery patterns (Riedel 1994c; Petrucci 2007). A cache of over 600 cattle horncores—associated with craft activities and therefore not included in this study—further suggest that the city was a centre of cattle horn and probably hide production (Riedel 1979). These types of deposits—rich in cattle bones with relatively standardised butchery modifications—are common across the Roman provinces, especially in towns and military sites, where they attest to specialised large-scale carcass processing and associated craft activities (e.g. Lepetz 1996; Maltby 2007; Groot 2016; Deschler-Erb 2017; Seetah 2018). In contrast to Roman times, large bone accumulations in pre- and protohistoric sites in northern Italy generally derived from the disposal and accumulation of domestic rubbish within settlements. Due to the size and number of assemblages available for this study, large cattle-dominated assemblages had a significant impact on NISP percentages, driving up the archaeological visibility of cattle in NISP metaanalyses in regions and on urban site types. Thus, it may have been this scale of cattle processing, and associated activities of butchery and material production (tanning, bone working, glue/oil production)—rather than sociocultural factors like meat preference—that divided urban and rural sites: when cattle-dominated deposits from Calvatone and Aquileia were excluded, urban 1, urban 2 and minor sites produced comparable and fairly balanced livestock percentages (cf. Online Supplement 2). Further zooarchaeological research and new assemblage analyses are needed to flesh out this conclusion: we were unable to include urban assemblages for the Roman Mid or Late phases in the southern Po Plain (e.g. from Piacenza, Modena, Bologna, or Ravenna) due to a lack of published zooarchaeological data, which is needed to appreciate animal consumption and processing on cities south of the River Po, and whether they aligned more closely with the pig-focused central Italian strategy (as suggested by Modena), the cattle-dominated trend of Aquileia, or another different model.

### Size change in domestic livestock

#### Cattle

After an initial increase between the Bronze and Iron Ages, cattle demonstrated a subsequent major size increase in Roman times (Figs. 4 and 5). The initial nature of this change during Republican times—a key transitional period—is difficult to characterise. One of the earliest Roman assemblages we considered—San Damaso, a minor agricultural site in the southern Po Plain—produced some of the largest Roman cattle included in this study. LSI length and width values from the site exceed all Iron Age samples, although a very large value from Etruscan Spina suggests an individual comparable in size. Biometric, isotopic and genetic results have provided evidence for cattle mobility and trade elsewhere in Western Mediterranean during the early Roman period (e.g. Minniti et al. 2014; Colominas and Edwards 2017; Nieto-Espinet et al. 2020), and changes in the circulation of these animals certainly would have occurred in Roman northern Italy. Considering the biometric evidence for pre-Roman livestock improvements, steps in this direction were probably underway during the Iron Age as well. Metacarpal measurements also suggest that the Iron Age was a moment of experimentation in cattle husbandry or with the adoption of new animal types. Compared to metacarpal distal breadth (Bd) measurements from the Bronze Age, which were roughly bimodal, Iron Age measurements produced a wider range and more uneven distribution of values (cf. Online Supplement 9). This distribution suggests greater intra-population variation in metacarpal biometry, possibly due to differences in sex ratios or castration practices, a greater diversity of cattle morphotypes, or even traction-related changes to metacarpal biometry (see Bartosiewicz 2008). Problematically, our data for the third and second centuries BC represent relatively marginal areas (e.g. Monte Bibele) or non-Etruscan regions of northern Italy with smaller Iron Age livestock (see Trentacoste et al. 2018), so it was not possible to trace high-resolution changes in the study region during the Republican period. Large early Roman cattle may therefore represent the evolution of a large cattle morphotype already present in the Etruscan Po Plain, or the introduction of a new large type with Roman colonisation of the area, or a combination thereof.

During the Roman Imperial period, the minimum, maximum and mean LSI values for cattle all increased. The smallest cattle recorded in Iron Age assemblages disappeared completely from the study area. Measurements from metacarpals provide limited insight into the representation of male and female animals in Roman herds, but Roman measurements fall across a similar range as prehistoric populations, indicating that both male and female cattle increased in size. The distribution of Roman metacarpal measurements illustrates a significant proportion of robust animals, comparable with male individuals from fifteenth-century Beja (Online Supplement 9). Previous discussions of cattle mortality patterns in Italy, which indicate a predominance of adult and elderly individuals, and documentation of traction-related pathologies have highlighted the role of Roman cattle in draught and agricultural labour (see MacKinnon 2004a; Salvadori 2015:265). The intensification and specialisation of production in large agricultural states in Roman times would have incentivised keeping large cattle and an increased use of oxen, as suggested by Classical sources (Varro,* On Agriculture* 1.20.1; Columella, *On Agriculture* 6.2.1).

After increasing in size in Republican to Imperial times, significant changes in LSI width values between the Mid and Late phases, as well as the Mid–Late and Late phases, suggest a decrease in the robustness of cattle sometime after the fourth century AD. Length values were more variable between Late sites, with very tall cattle at Aquileia (fourth–fifth century AD) and much shorter animals at Udine (fourth–seventh century AD) and Torcello (fifth–seventh century AD). This distinction may suggest that decreases in livestock height date to the very end of the chronology considered here, i.e. the sixth–seventh century AD, thus coinciding with broader socioeconomic reorganisation related to the Gothic Wars or Lombard invasion. Alternatively, it may reflect differences in site type or context: Aquileia, an important city and imperial seat, would have had different supply requirements and economic networks than the island settlement of Torcello, where smaller or female animals may have been preferred. Still, the small size of cattle at Udine and Torcello align with broader Italian trends that register a decrease in the height of cattle during the fourth to sixth centuries AD (Salvadori 2015, 2019). A decrease in cattle LSI width values, although not statistically significant, has also been documented in Rome during this period (De Grossi and Minniti 2017). Ultimately, with only three assemblages for the period, we are unable to establish the precise timing or nature of size developments, but the large size of cattle across the Roman Mid–Late phase suggests that any diminution was a feature of the latest assemblages considered in this study, resulting from the progressive reorganisation of the rural landscape over several centuries and/or the ultimate collapse of agrarian system in the sixth century AD (Banaji 2012; Castrorao Barba 2014; Forin 2017).

The development of large-scale cereal production within a market-orientated villa economy of Imperial Italy, followed by the emergence of more locally oriented, diverse, and self-sufficient means of food production, would have had a significant impact on cattle husbandry, particularly the exploitation of draught oxen in some areas (Rottoli 2014; Salvadori 2015:92–97; Varro, *On Agriculture* 1.20). Considering the general continuity in cereal preference in northern Italy, which focused on cultivation of barley and wheats from the late Iron Age through Late Antiquity (Rottoli 2014; Bosi et al. 2019; Bosi et al. 2020), greater use of large oxen during the Roman period points to a change in how staple crops were produced. For example, cultivation of larger plots compared to prehistoric practices would have incentivised animal over human labour (Bogaard et al. 2019). The Iron Age/Etruscan period may have seen moves toward similar agricultural developments that encouraged exploitation of large cattle, especially in more intensively used and urbanised landscapes within the Po Plain (e.g. Govi 2014). These agricultural systems relaxed gradually over Late Antiquity and post-classical times, eventually giving way to more local, small-scale and diverse food systems (Salvadori 2011; Squatriti 2013; Rottoli 2014). If accompanied by a shift towards human labour in farming practices (Rottoli 2014), it would incentivise keeping smaller, less demanding cattle—rather than large oxen—or cows that could be used for dairying as well as light work (Salvadori 2015; Varro, *On Agriculture* 1.20.4 ).

#### Sheep and goats

Sheep also increased in size in Roman times, and where sufficient data was available, goats produced similar trends. However, their development differed from that of cattle: size increases in the Roman period were less exceptional compared to Iron Age improvements, and developments were more visible in widths than in heights. Differences between Iron Age and Roman LSI means from sheep were smaller than those between the prehistoric periods; M3 measurements reinforce this continuity. While the mean of LSI values increased, the largest Roman sheep were comparable in height to large Iron Age animals, excluding a few exceptional individuals. These significant developments in the Iron Age/Etruscan period indicate a reorganisation of sheep management well before Roman control (Trentacoste et al. 2018), likely encouraged by increasing centralisation and specialisation in textile production during the Iron Age (Gleba 2008; Trentacoste 2020). Textile production, and sheep exploitation in support of it, may have been particularly developed in the southern Po Plain, where there is evidence for a livestock and wool market at the Campi Macri near Mutina in the Celtic period (Ortalli 2012).

Roman animals, however, were notably more robust than Iron Age sheep, and the bimodal distribution of metacarpal Bd measurements illustrates the emergence of two groups: one comparable to Iron Age sheep and a second significantly more robust type. This distribution of distal metacarpal values is very different from those of the prehistoric periods, suggesting an increase in the management of rams and castrates in Roman compared to prehistoric herds. The Roman author Columella describes how fine-wooled sheep in southern Italy were raised in flocks with more castrated male animals than coarser-wooled varieties, due to the superior quality—and higher prices—of fleeces obtained from wethers (Columella, *On Agriculture* 7.2.3–4). While zooarchaeological evidence from age profiles suggests a significant variability in management strategy across northern Italian assemblages (MacKinnon 2004a), Cisalpine Gaul is one of the regions repeatedly cited by Roman authors as a key area of wool production (MacKinnon 2004b; Busana et al. 2012). Northern Italy was renowned for the quality of its fleeces, which came in different colours and levels of fineness suited to different purposes (Columella, *On Agriculture* 7.2.3; Pliny, *Natural History* 8.190). Sheep from Altinum (Altino), Mutina (Modena), Parma, and the vicinity of the Panaro River are named as especially valuable on account of their wool, with Padova also recognised as a centre of production (Columella, *On Agriculture* 7.2.3–4; Strabo, *Geography* 5.1.12). Towns in northern Italy were also renowned for production of different types of textiles, and numerous inscriptions attest to the many individuals and associations involved in their creation (Flohr 2013; Liu 2013) as part of an export-driven economy (Flohr 2016). In this context, the composition of sheep herds, especially those specialised in the breeding of finer-wooled varieties, may have shifted to include more male animals. Replacement or interbreeding of local animals with new, more robust types for reasons of fleece quality as well as other motivations is also possible, alongside changes in sex ratios.

Like in cattle, the smallest sheep all but disappeared from the published Roman data, and a decrease in width—but not length values—was noted in the latest sites considered in the study. This trend may suggest a reconfiguration of sheep management after the fourth century AD, possibly around the sixth century AD, although with few sites clear conclusions are not possible. Broad analysis of Italy as a whole indicates relative continuity in sheep biometry from Late Antiquity through the early Middle Ages, with notable size diminution around the tenth–eleventh centuries AD (Salvadori 2015); the trend is also visible in biometric data from Verona in northern Italy (Riedel 1994a, b). In Rome, however, sheep/goat LSI length values in fact increased between Imperial and Late Antique times (De Grossi and Minniti 2017). This development might have a parallel in the significant increase in LSI width values found between Roman Mid and Mid–Late (as well Early and Mid–Late) phases in this study, but with the few well dated assemblages, it is difficult to establish the timing of and therefore relationship between these trends. Further quantitative comparisons with medieval data are needed to contextualise these changes, and the pace of change in northern Italy compared to broader Italian trends.

#### Pigs

Compared to domestic bovids, pigs followed a different trajectory. Pigs decreased in height over later prehistory, while LSI width values remained comparatively unchanged. Only in the Roman period did they become taller and more robust, in a process that appears to have been gradual. MacKinnon (2001, 2004a) also recorded minor increases in mean pig withers height between Republican and Late Antique periods. Our results corroborate these conclusions, with mean values increasing progressively, with significant differences compared to IA2 values becoming visible in the Roman Mid–Late and Late phases (see Fig. 10; Online Supplement 8). Measurements from pig bones alongside iconographic depiction of pigs different in colour and hairiness has led to the suggestion of the presence of two ‘breeds’ of pig in Roman Italy (MacKinnon 2001): a smaller, dark, bristly pig (60–75 cm withers height) and large, light-coloured, smooth pig (c. 80 cm withers height). In this study, large outliers were consistent with wild *Sus* common in the prehistoric periods, suggesting that many large Roman specimens identified as domestic pigs are potentially wild boars rather than domestic animals. Still, hybrids and imported domestic pigs may be concealed within the sample: comparison of pig biometry in the Western provinces suggests that pigs in Roman Italy were markedly smaller than contemporaneous animals in other parts of the Empire (Frémondeau et al. 2017).

In contrast to the size increase registered across the Roman period in northern Italy, biometric data from central Italy suggest a different trend, with increases in pig size between Republican and Imperial times, but decreases in pig LSI width values (but no change in length values) during Late Antiquity (De Grossi and Minniti 2017); however, by the seventh–eighth century AD, pigs in Rome were markedly larger than their Imperial counterparts (Albarella et al. 2019). If the divergent patterns in Late Antique north and central in pig biometry can be taken as representative—and not simply an artefact of small sample size or site type—they may reflect broader economic and environmental developments. Firstly, the decline in Rome as a major consumer market and reconfiguration of northern Italy as a seat of imperial power and military activity would have had differing impacts on the two regions. Pork consumption in Rome progressively declined from the fifth century AD (De Grossi and Minniti 2017), which would impact other areas of the peninsula where these animals were produced (Barnish 1987; Belli Pasqua 1995). Communities in the north may have stopped supplying large pigs or preserved pork cuts to Rome, or otherwise reorganised production in response to new supply demands and increased military presence in the region. Security concerns in the wake of invasions could also promote greater control over animals through more local management, enclosure, or stall-feeding, potentially facilitated by the ruralisation of urban environments. Changes to the composition of the countryside may also have had an impact. If communities continued to utilise extensive free-range pig herding strategies, greater contact and inbreeding with larger wild boar might promote Late Antique increases in body size (Albarella et al. 2019). Forest regrowth and less intensive land usage patterns would have offered increased opportunities for such meetings, while also increasing the availability of grazing environments for wild foods (e.g. Squatriti 2013)—feeding patterns that may also have improved pig diets. Studies of dietary isotopes would shed much light on these hypotheses (e.g. Hamilton and Thomas 2012), as would more detailed examination of pig mortality patterns.

## Conclusions: continuities and changes in livestock production in Roman north Italy

The evolution of taxon representation and animal biometry documented in this study this point to both continuities and changes in the management of livestock in northern Italy. Continuities can be seen between the late Iron Age and Roman times in the progressive size increases in cattle, sheep and goats, alongside—in Friuli and the South study area—the continuation of trends in livestock abundance established in the late Iron Age. These developments suggest that the first steps towards a more dynamic and integrated livestock economy predate Roman political annexation of the region, and result less from new Roman technological innovation or a new Roman interest in large animals, but from the ability of different socioeconomic structures to facilitate new forms of animal and land management (Lepetz 1997; Valenzuela-Lamas and Albarella 2017; Duval and Clavel 2018). New patterns of settlement organisation and forms of social connectivity (e.g. Fulminante 2014; Cavazzuti et al. 2019a) and increasingly complex and integrated economic systems (Morel 2007; Sestieri 2008; Nijboer 2017) would have encouraged this pre-Roman reorganisation of livestock management (Trentacoste 2020). Drawing parallels from Roman examples, new mobility regimes and exchange strategies probably helped catalyse these early developments in animal size (Valenzuela-Lamas 2020), potentially alongside new foddering regimes and breeding strategies (Méniel 1996; Kron 2002) that were adjusted alongside broader changes to farming strategies. Roman annexation and integration with the marketised supply systems of the Roman Republic and Empire brought about greater connectivity, supporting a further suite of changes to livestock production (Albarella et al. 2008; Valenzuela-Lamas and Albarella 2017), and—in northern Italy—further development of existing trends in sheep, goat and cattle size.

Alongside this evidence for continuities in livestock management, zooarchaeological analyses illustrate significant changes in how livestock were managed and processed during the Roman period. Special cattle bone deposits dominate regional NISP analyses, illustrating new large-scale and standardised modes of carcass processing. The shortest cattle and sheep disappear from the zooarchaeological data and changes in metacarpal biometry suggesting further reconfiguration of the farming system and potentially specialisation in livestock exploitation. Pigs reversed their prehistoric trend towards size diminution and gradually increased in size, suggesting a change to their management despite the continued use of extensive husbandry practices (see MacKinnon 2001). Divergent patterns are also suggested in regional trends in livestock body size during Late Antiquity, when northern and central Italy appear to follow different courses at the end of the period considered here—results that, if not an artefact sample size and site chronologies, may reflect of meaningful changes related to the socioeconomic and political trajectories of these regions during Late Antiquity, related to, for example, greater militarisation, taxation and political division the of peninsula into *Annonaria* (geared at supplying the army) and *Suburbicaria* (supplying Rome) diocese (Christie 2006:65).

Thus zooarchaeological evidence illustrates both new trajectories and acceleration of established prehistoric trends in livestock exploitation between late prehistory and Late Antiquity. These conclusions, however, raise further questions on people’s changing relationships with the same key animal species—relationships at the heart of the most basic and essential area of ancient production: farming. Further research has the potential to shed new light on agriculture practices, particularly through new zooarchaeological and archaeobotanical assemblage studies, metadata analyses considering additional variables like age data, comparisons with central Italy and circum-Alpine zones, and bioarchaeological techniques with the potential to provide details on where and how plants and animal were managed. Approaches informed by landscape variables might also help unravel complex sub-regional variation in features like NISP values (e.g. Brandolini and Carrer 2020), especially in a region where lowland zones had increasingly active relationships with upland areas (and their complementary ecologies) from late prehistory. Current data demonstrate that socioeconomic changes in the Iron Age and political unification under the Roman Republic and Empire had clear, and sometimes similar, impacts on farming strategies. Further research will continue to shine light on the specific changes in human behaviour that underlie these trends, and the decisions that shaped agriculture at the heart of the Empire in Roman Italy.

## Supplementary Information

Supplement 1 Site list with region, location, NISP, number of measurements, and references (CSV 44 kb)

Supplement 2 Livestock representation by site type (PNG 108 kb)

Supplement 3 Results of chi square tests for livestock NISP (CSV 2 kb)

Supplement 4 Summary statistics for LSI values by period (CSV 1 kb)

Supplement 5 Summary statistics of mandibular third molar (M3) length and width (mm) by period (CSV 788 bytes)

Supplement 6 Summary statistics of LSI values by for phases within the Roman–Late Antique period (CSV 1 kb)

Supplement 7 Results of Mann-Whitney U tests comparing LSI values by period (CSV 1 kb)

Supplement 8 Results of Mann-Whitney U comparing LSI values for phases within the Roman–Late Antique period (CSV 5 kb)

Supplement 9 Cattle metacarpal biometry (PNG 168 kb)

Supplement 10 Sheep metacarpal biometry (PNG 156 kb)

Supplement 11 Pig metacarpal biometry (PNG 85 kb)

## Data Availability

Not applicable.
