# Supplementary figures and images for: New trajectories or accelerating change? Zooarchaeological evidence for Roman transformation of animal husbandry in Northern Italy
Source: Archaeol Anthropol Sci. 2021 Jan 15;13(1):25. doi: 10.1007/s12520-020-01251-7 (PMC7810668; doi:10.1007/s12520-020-01251-7)

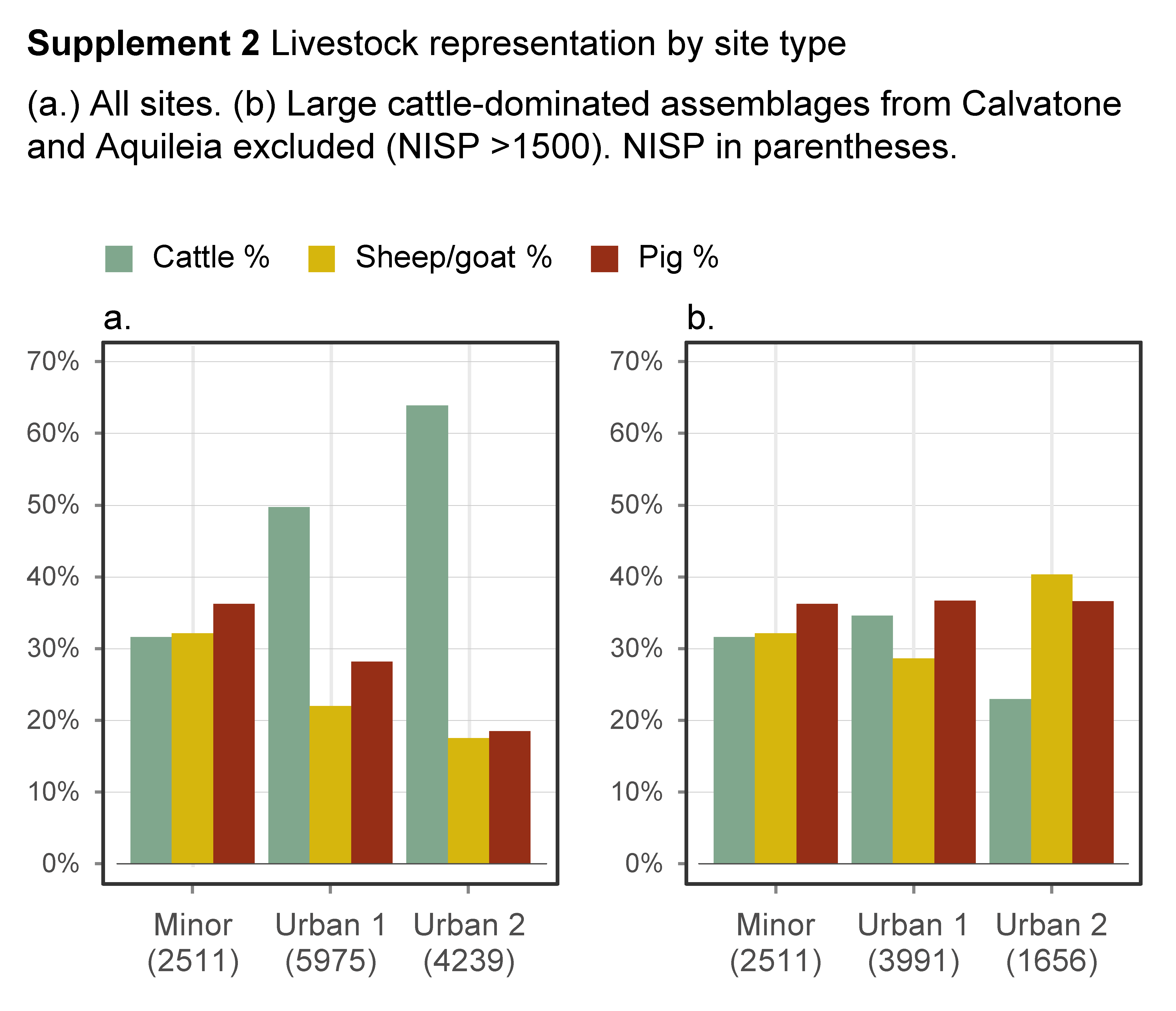

Supplement: Supplementary file 2 — Livestock representation by site type (PNG 108 kb) [file 12520_2020_1251_MOESM2_ESM.png]

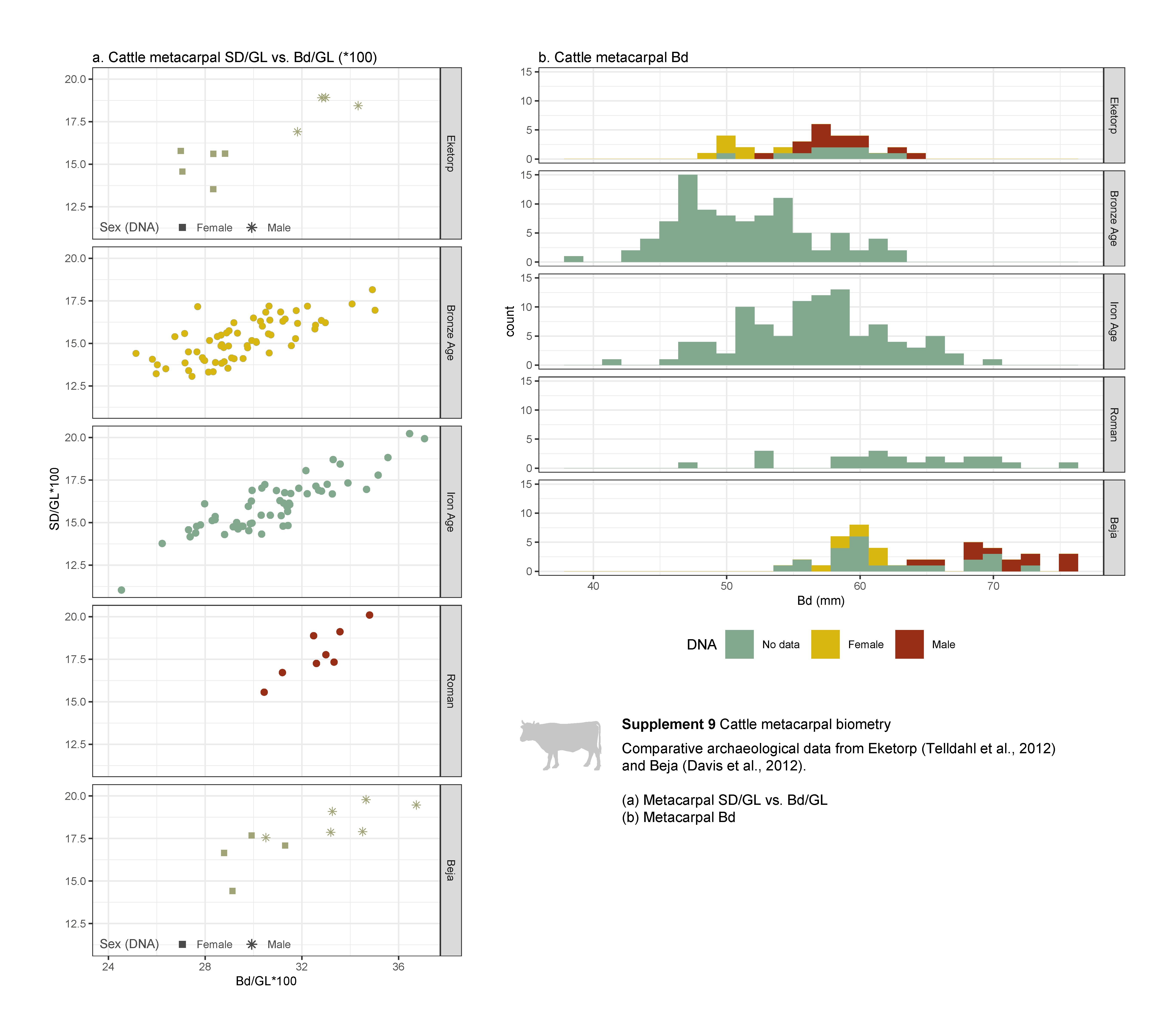

Supplement: Supplementary file 9 — Cattle metacarpal biometry (PNG 168 kb) [file 12520_2020_1251_MOESM9_ESM.png]

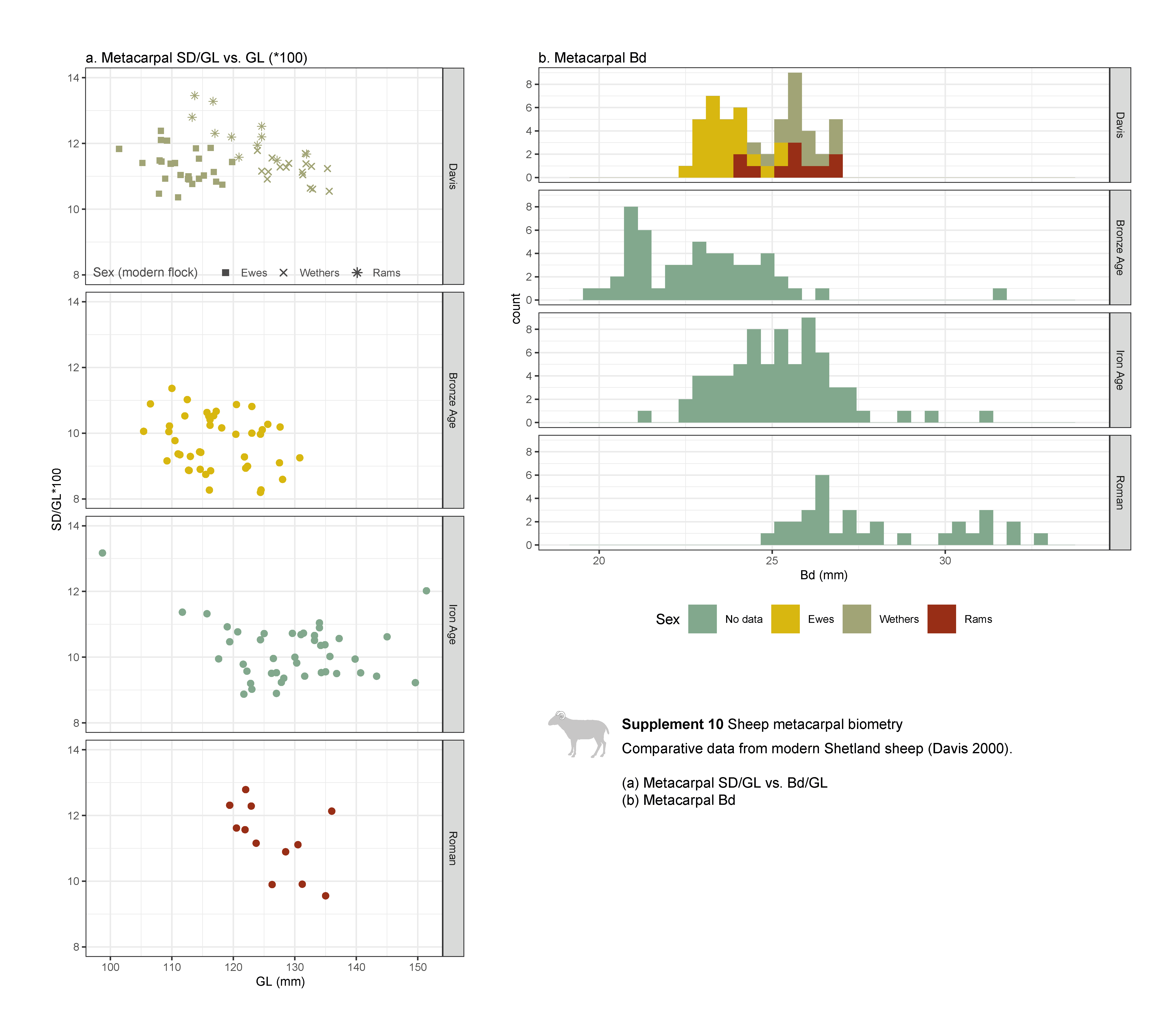

Supplement: Supplementary file 10 — Sheep metacarpal biometry (PNG 156 kb) [file 12520_2020_1251_MOESM10_ESM.png]

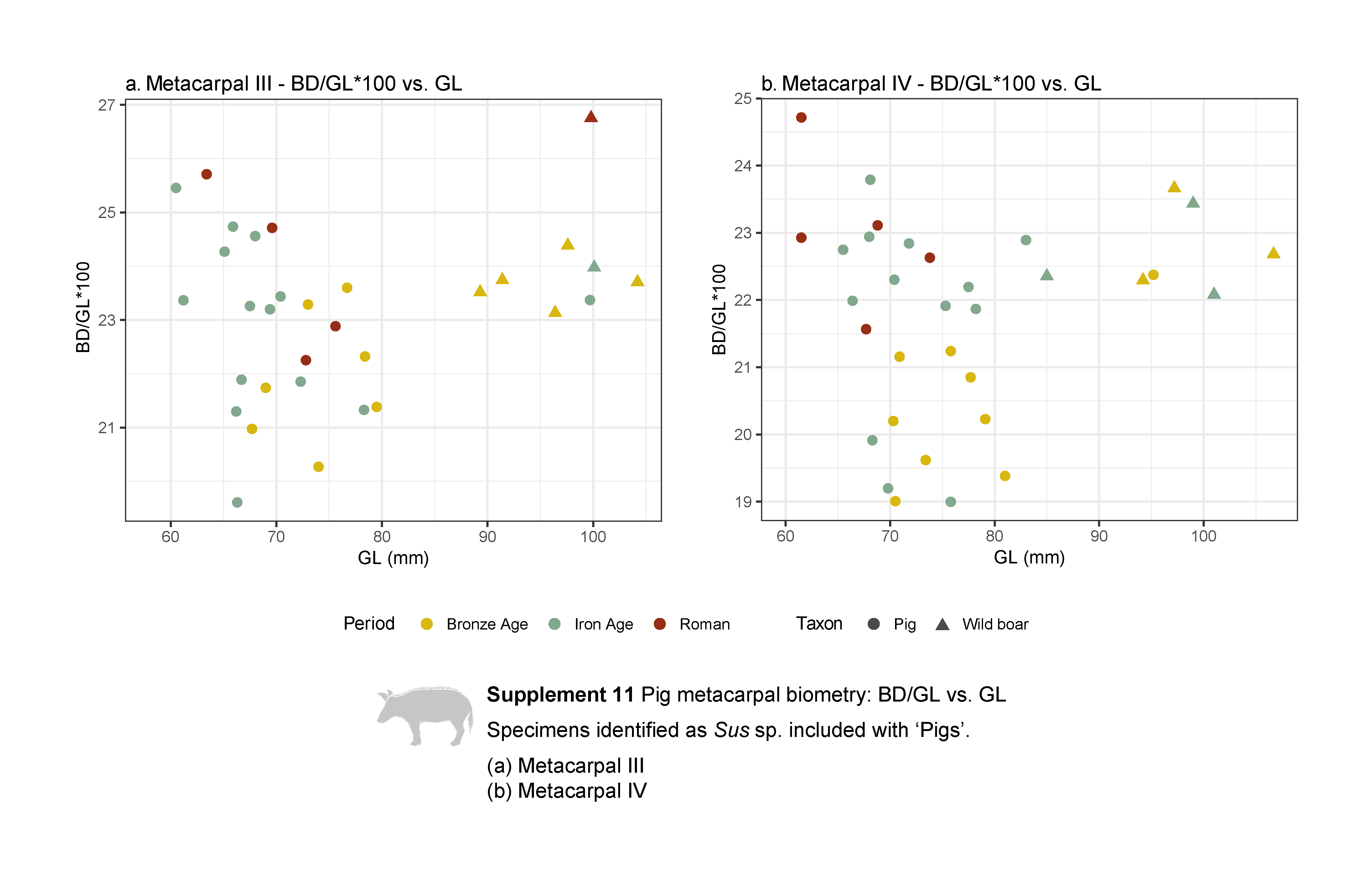

Supplement: Supplementary file 11 — Pig metacarpal biometry (PNG 85 kb) [file 12520_2020_1251_MOESM11_ESM.png]
